# Qianggu concentrate: unlocking bone protection power via antioxidative SIRT1/NRF2/HO-1 pathways in type 2 diabetic osteoporosis

**DOI:** 10.3389/fphar.2024.1426767

**Published:** 2024-08-08

**Authors:** Kaili Wang, Xiang Dang, Yanyan Wang, Qing Yang, Tingting Zhang, Peng Yang, Ling Yuan, Rongming Xu, Yuqi Dang, Yi Nan

**Affiliations:** ^1^ College of Traditional Chinese Medicine, Ningxia Medical University, Yinchuan, China; ^2^ Department of Endocrinology, Yinchuan Hospital of Traditional Chinese Medicine, Affiliated with Ningxia Medical University, Yinchuan, China; ^3^ College of Pharmacy, Ningxia Medical University, Yinchuan, China

**Keywords:** traditional Chinese medicine, diabetic, osteoporosis, oxidative stress, osteogenic differentiation

## Abstract

**Background:**

Qianggu Concentrate (QGHJ), a traditional Chinese medicine, is extensively used to treat Type 2 Diabetic Osteoporosis (T2DOP). Despite its widespread use, research on its therapeutic mechanisms within T2DOP is notably scarce.

**Objective:**

To explore QGHJ’s osteoprotection in T2DOP rats and BMSCs, focusing on the antioxidant activation of SIRT1/NRF2/HO-1 and NRF2 nuclear migration.

**Methods:**

QGHJ constituent analysis was performed using UPLC-HRMS. Safety, bone-health efficacy, and glucose metabolic effects in T2DOP rats were evaluated via general condition assessments, biomarker profiling, micro-CT, biomechanics, staining methods, and ELISA, supplemented by RT-qPCR and Western blot. BMSCs’ responses to QGHJ under oxidative stress, including viability, apoptosis, and osteogenic differentiation, were determined using CCK-8, flow cytometry, ALP/ARS staining, and molecular techniques. The modulation of the SIRT1/NRF2/HO-1 pathway by QGHJ was explored through oxidative stress biomarkers, immunofluorescence, and Western blot assays.

**Results:**

UPLC-HRMS identified flavonoids, monoterpenes, and isoflavones as QGHJ’s key compounds. *In vivo*, QGHJ proved safe and effective for T2DOP rats, enhancing bone mineral density, microenvironment, and biomechanical properties without impairing vital organs. It modulated bone markers PINP, TRACP 5b, RUNX2 and PPARγ, favoring bone anabolism and reduced catabolism, thus optimizing bone integrity. QGHJ also regulated glycemia and mitigated insulin resistance. *In vitro*, it preserved BMSCs’ viability amidst oxidative stress, curbed apoptosis, and fostered osteogenesis with regulated RUNX2/PPARγ expression. Mechanistic insights revealed QGHJ activated the SIRT1/NRF2/HO-1 pathway, augmented NRF2 nuclear translocation, and enhanced the antioxidative response, promoting bone health under stress.

**Conclusion:**

In T2DOP rat and BMSCs oxidative stress models, QGHJ’s bone protection is anchored in its antioxidative mechanisms via the SIRT1/NRF2/HO-1 pathway activation and NRF2 nuclear translocation.

## Graphical Abstract

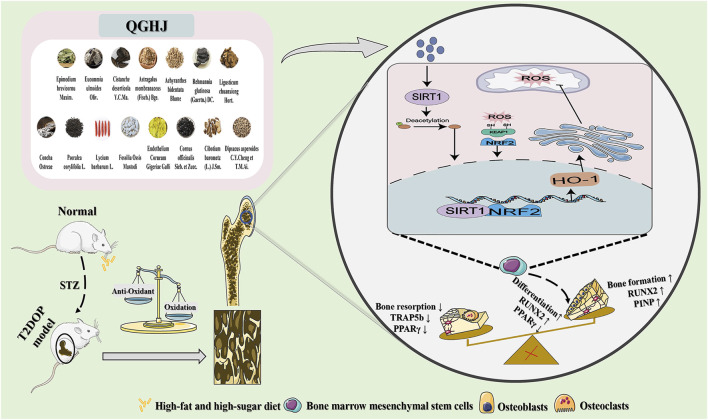

## 1 Introduction

Type 2 diabetic osteoporosis (T2DOP), a severe skeletal complication of type 2 diabetes mellitus (T2DM), is characterized by bone loss and increased fracture risk. Diabetes significantly compromises bone health in millions of patients globally (Schacter and Leslie, 2021). Research suggests that up to 60% of T2DM patients develop osteoporosis (Zhang et al., 2019). These findings underscore the pressing need for effective strategies to manage T2DOP. Current treatment approaches for T2DOP combine lifestyle interventions with hypoglycemic and anti-osteoporotic medications. Lifestyle modifications improve glycemic control and bone health but require long-term adherence for optimal efficacy. Metformin and glucagon-like peptide-1 receptor agonists (GLP-1RAs) show positive or neutral effects on skeletal health while lowering blood glucose (Josse et al., 2017). Metformin influences bone metabolism by activating AMP-activated protein kinase in cultured bone marrow progenitor cells and osteoblasts (Chen et al., 2017). A recent meta-analysis associated metformin treatment for type 2 diabetes with reduced fracture risk (Hidayat et al., 2019). Liraglutide, a GLP-1RA, promotes osteoblast differentiation and bone formation by activating PI3K/AKT and cAMP/PKA signaling pathways via GLP-1 receptor binding (Ming-Yan et al., 2019). However, thiazolidinediones, sulfonylureas, and SGLT-2 inhibitors should be avoided in T2DOP treatment as they may decrease bone mineral density and increase fracture risk (Rajagopalan and Brook, 2017; Jackson and Moseley, 2020; Napoli et al., 2021). Various anti-osteoporosis medications effectively improve bone mineral density and reduce fracture risk (Tabacco and Bilezikian, 2019). These include calcium and vitamin D supplements, bisphosphonates, and parathyroid hormone analo gs. These medications, however, have limitations and potential side effects. Calcium and vitamin D supplements alone have limited efficacy. Bisphosphonates may cause gastrointestinal discomfort and, rarely, serious adverse effects like atypical femoral fractures and jaw osteonecrosis with long-term use (Silverman et al., 2018). Parathyroid hormone analogs have limited treatment duration and high costs. Traditional Chinese medicine (TCM) offers a unique, holistic approach to disease management, focusing on restoring overall body balance (Liu et al., 2020). Qianggu Concentrate (QGHJ), a TCM formula, shows promise in T2DOP management. QGHJ’s multi-component, multi-target mechanism potentially addresses both diabetic and osteoporotic aspects of T2DOP simultaneously.

QGHJ, a traditional Chinese medicine formula, has long been used to treat T2DOP by invigorating the spleen, tonifying the kidney, and strengthening tendons and bones. QGHJ is based on traditional Chinese medicine theory, which asserts that the kidney regulates bone health (Ju et al., 2014; Yang et al., 2022; Zhu et al., 2022) and the spleen controls metabolism and musculoskeletal function (Yang and Jia, 2013; Xiang et al., 2016). QGHJ incorporates traditional wisdom from the classical text “Jingyue Quanshu”, which describes the Yougui Pill. Key herbs from this pill, such as Eucommia ulmoides Oliv., Cornus officinalis Sieb. et Zucc., and Lycium barbarum L., have been traditionally used for their bone-strengthening properties (Li et al., 2018; Gu et al., 2019). Preliminary clinical studies have demonstrated QGHJ’s efficacy in alleviating knee pain, reducing blood glucose levels, increasing bone mineral density (BMD), and improving bone metabolism markers (Yang et al., 2015; Li W. et al., 2023). The QGHJ formulation comprises Eucommia ulmoides Oliv. [Eucommiaceae; bark] 5 g, Astragalus membranaceus (Fisch.) Bge. [Fabaceae; root] 5 g, Cornus officinalis Sieb. et Zucc. [Cornaceae; fruit] 3 g, Rehmannia glutinosa (Gaertn.) DC. [Orobanchaceae; tuber] 8 g, Lycium barbarum L. [Solanaceae; fruit] 5 g, Achyranthes bidentata Blume [Amaranthaceae; root] 3 g, Epimedium brevicornu Maxim. [Berberidaceae; leaf] 6 g, Fossilia Ossis Mastodi [fossil; bone] 5 g, Concha Ostreae [Ostreidae; shell] 5 g, Processed Endothelium Corneum Gigeriae Galli [Phasianidae; inner lining of chicken gizzard] 3 g, Cibotium barometz (L.) J. Sm. [Cibotiaceae; rhizome] 5 g, Psoralea corylifolia L. [Fabaceae; seed] 4 g, Dipsacus asperoides C.Y.Cheng et T.M.Ai. [Caprifoliaceae; root] 4 g, Ligusticum chuanxiong Hort. [Apiaceae; rhizome] 4 g, and Cistanche deserticola Y.C.Ma. [Orobanchaceae; stem] 4 g. QGHJ incorporates herbs traditionally used to enhance bone strength and kidney function (E. ulmoides, C. officinalis, A. bidentata, E. brevicornu, P. corylifolia, D. asperoides), as well as herbs known to nourish yin, promote fluid balance, and strengthen spleen function to alleviate diabetes symptoms (A. membranaceus, R. glutinosa, L. barbarum). Pharmacological studies have demonstrated synergistic effects among QGHJ herbs, enhancing anti-T2DOP efficacy. E. brevicornu combined with P. corylifolia has been shown to increase flavonoid bioavailability, enhancing anti-osteoporotic effects (Wang et al., 2024). C. officinalis and A. bidentata have been found to promote osteogenic differentiation by upregulating ALP and Runx2 genes (Park et al., 2020a). C. officinalis combined with R. glutinosa has been reported to maintain serum insulin levels and reduce diabetic symptoms in mice (Lv et al., 2016). A. membranaceus and R. glutinosa have been shown to decrease ROS levels in rats with diabetic foot ulcers, mitigating oxidative stress (Lau et al., 2012). Individual herbs in QGHJ have demonstrated specific therapeutic effects. Animal research has demonstrated that E. ulmoides extract prevented bone loss and bone turnover in rats (Pan et al., 2014), while P. corylifolia extract stimulated osteosarcoma cell proliferation and increased ALP activity, indicating potential anti-osteoporotic effects (Cai et al., 2021). A. membranaceus has been found to significantly reduce blood glucose levels in diabetic models (Liu et al., 2024). E. brevicornu has shown multifaceted pharmacological benefits, including anti-inflammatory and hypoglycemic effects (Yang et al., 2020). Furthermore, the aqueous extract of R. glutinosa has been reported to significantly increase femur and lumbar vertebrae BMD and decrease serum ALP levels (Lim and Kim, 2013).

Oxidative stress (OS), caused by excessive free radicals, contributes significantly to cellular damage (Sies et al., 2017). In T2DM patients, chronic hyperglycemia exacerbates oxidative stress, impairing pancreatic β-cell function and accelerating disease progression (Lotfy et al., 2017). Reactive oxygen species (ROS), particularly hydrogen peroxide and superoxide anions, directly impact bone metabolism by enhancing osteoclast activity and inhibiting osteoblast function (Zhou et al., 2021a). The SIRT1/NRF2/HO-1 signaling pathway plays a crucial role in protecting against oxidative damage (El-Shitany and Eid, 2019). Activation of the Nrf2/HO-1 axis reduces ROS accumulation and mitochondrial dysfunction, promoting bone marrow stromal cell (BMSC) osteogenesis (Jin et al., 2022). Therefore, therapeutic strategies targeting oxidative stress pathways are promising for addressing T2DOP-associated bone metabolism disorders.

**FIGURE 1:**
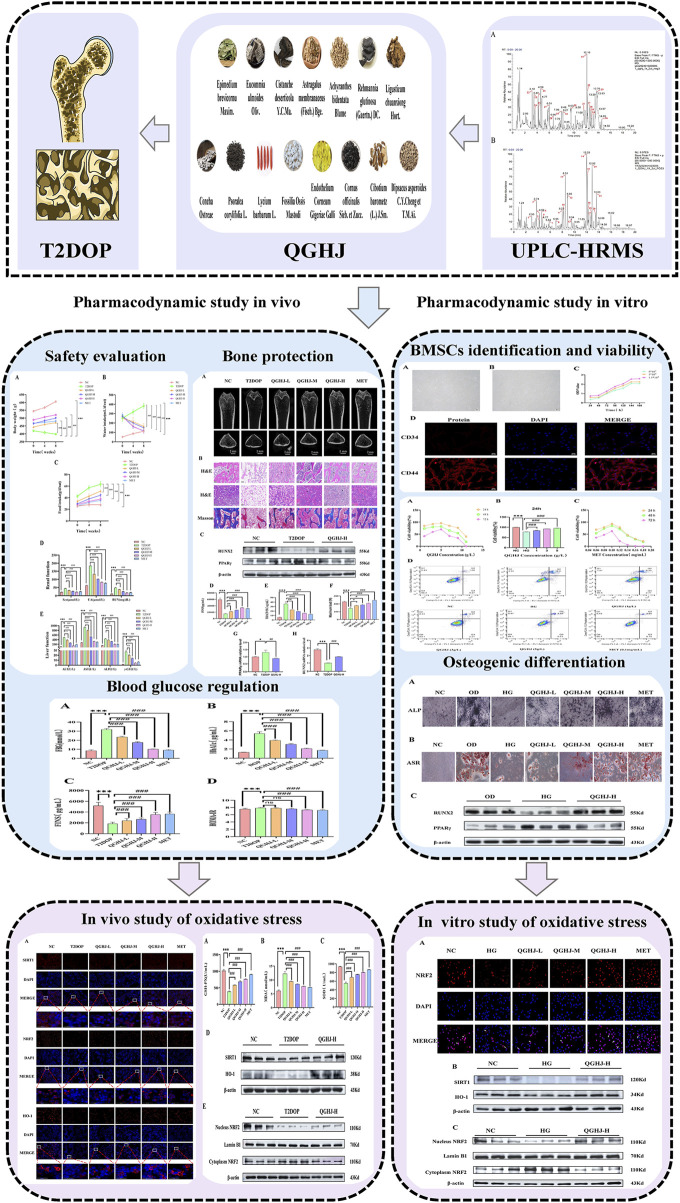
Flow chart.

In this study, we used UPLC-HRMS to identify QGHJ’s active components for treating T2DOP, providing a foundation for exploring its mechanism of action. We validated QGHJ’s effects on bone protection and glucose metabolism in a T2DOP rat model and investigated its impact on BMSC viability and osteogenic differentiation under oxidative stress *in vitro*. Moreover, mechanistic experiments were conducted to measure oxidative stress markers and the activation and nuclear translocation of the SIRT1/NRF2/HO-1 pathway (Figure 1).

## 2 Materials and methods

### 2.1 Experimental drugs

Individual herbs comprising QGHJ were sourced from Yinchuan Traditional Chinese Medicine Hospital. The QGHJ was then prepared following standard protocols for Traditional Chinese Medicine in medical institutions. QGHJ preparation involved immersing herbs in water (1:8 w/v) for 2 h, followed by 2 h of boiling. The decoction was then filtered, centrifuged, concentrated, and adjusted to a final concentration of 0.33125 g/mL. For cell experiments, the prepared QGHJ was further centrifuged (4°C, 3,500 rpm, 15 min) and filtered through a 0.22 μm membrane.

Metformin hydrochloride (MET) was employed as a positive control in this study. For *in vivo* animal experiments, extended-release tablets (MET, C192204223, Ouyi Pharma, China) were utilized. *In vitro* assays used Metformin hydrochloride (HY-17471A, MedChemExpress, USA).

### 2.2 UPLC-HRMS analysis of QGHJ

The QGHJ supernatant, following centrifugation and vacuum lyophilization, was resuspended in 40% methanol and thoroughly mixed. This mixture was then centrifuged (16,000 g, 4°C, 15 min), and the resulting supernatant was collected for analysis. Chromatographic separation employed a Vanquish UHPLC system (Thermo Fisher Scientific, Germany) equipped with an ACQUITY UPLC HSS T3 column (2.1 mm × 100 mm, 1.8 µm). The column temperature was maintained at 35°C with a flow rate of 0.3 mL/min. The mobile phase included 0.1% formic acid in water (A) and acetonitrile (B). The elution began with 2% solvent A for 17 min, linearly progressed to 98% solvent B over 17 min, then reversed to 95% solvent A for the next 17 min, shifted to 5% solvent B for 17.2 min, concluding with 95% solvent A and 5% solvent B each for 20 min. Mass spectrometric analysis was performed using a Q-Exactive HFX mass spectrometer (Thermo Fisher Scientific, Germany) coupled with the UHPLC system. Samples were ionized via electrospray in both positive (3,800 V) and negative (3,500 V) modes. The mass spectrometer operated in Full-MS/dd-MS2 mode with resolutions of 60,000 and 15,000 for full and data-dependent secondary scans, respectively. MS/MS spectra were acquired for the top 10 MS1 ions using stepped normalized collision energies (20, 40, and 60). The mass-to-charge ratio scan range was 90–1,300. Data analysis was performed using XCMS software, with results compared to a local high-resolution MS database for Chinese herbs. Component identification criteria included mass accuracy < 25 ppm and fit score > 0.7 to ensure reliability.

### 2.3 Assessment of QGHJ’s pharmacodynamic effects on T2DOP rats

#### 2.3.1 Experimental animals

Forty male Sprague-Dawley (SD) rats were obtained from the Animal Center of Ningxia Medical University (license number: SYXK (Ning) 2020–0001). The rats were maintained in the animal laboratory of Ningxia Medical University under controlled conditions: (21 ± 1)°C, 41%–51% humidity, (20 ± 2) Pa pressure difference, and a 12-h light/dark cycle. The rats were housed in groups of three per cage in a specific pathogen-free (SPF) environment with *ad libitum* access to food and water. The study protocol was approved by the institutional ethical committee (approval number: IACUC-NYLAC-2022–098) and adhered to the guide for the care and use of Laboratory Animals (Research, 1996).

#### 2.3.2 Animal model and grouping

To establish the T2DOP rat model, animals were initially fed a high-sugar, high-fat diet (HSHFD) for 4 weeks to induce insulin resistance (Zhang J. J. et al., 2022). The HSHFD (Boaigang-1135DM, Beijing Boaigang Biotechnology Co., Ltd., China) comprised 65% standard rat chow, 10% lard, 20% sucrose, 2.5% cholesterol, 1% sodium cholate, 1% mineral mixture, and 0.5% cellulose mixture. Subsequently, type 2 diabetes mellitus (T2DM) rat model (Xie et al., 2018) was induced by an intraperitoneal injection of 1% streptozotocin (STZ) at 30 mg/kg (BJBAG2001, Beijing Boaigang Biotechnology Co., Ltd., China) (Liang et al., 2021). The T2DM model was confirmed by fasting blood glucose levels ≥ 16.7 mmol/L and consistent diabetic symptoms (Liu S. et al., 2022). To induce osteoporosis, T2DM rats were maintained on the HSHFD for an additional 8 weeks (Liu et al., 2020). Osteoporosis was confirmed by reduced bone density (Samsulrizal et al., 2021).

Male SD rats were randomly divided into six groups (n = 6). Normal control (NC) group: rats fed a standard diet without interventions; T2DOP control group (T2DOP): rats fed the HSHFD and injected with 1% STZ (30 mg/kg), receiving no treatment; Metformin positive control group (MET): T2DOP rats treated with metformin (200 mg/kg/day) (Chen et al., 2022; Lin et al., 2023); QGHJ treatment groups: T2DOP rats treated with low (QGHJ-L, 1.7 g/kg/day), medium (QGHJ-M, 3.4 g/kg/day), or high (QGHJ-H, 6.8 g/kg/day) doses of QGHJ. QGHJ dosages were calculated based on clinically equivalent doses using a human-to-rat conversion factor (Jia et al., 2022). The formula applied was: rat dosage (QGHJ, g/kg) = (clinical dose × 0.33125 g/mL)/60 kg × 6.2. Clinical doses of 50, 100, and 200 mL corresponded to rat dosages of 1.7, 3.4, and 6.8 g/kg for low, medium, and high doses, respectively. QGHJ was administered via gavage to QGHJ-L/M/H groups at 5, 10, and 21 mL/kg, respectively. Metformin, dissolved in distilled water, was given to the MET group at 10 mL/kg. NC and T2DOP groups received equivalent volumes of distilled water. All treatments were administered twice daily for 8 weeks.

#### 2.3.3 Animal sample collection

Rats were anesthetized with intraperitoneal injection of pentobarbital sodium (2% w/v, 40 mg/kg). Blood was allowed to clot at 4°C for 2 h, and centrifuged (4,500 rpm, 15 min) to obtain serum. Euthanasia was performed using isoflurane overdose (5% in 95% oxygen). Femurs were fixed in 4% paraformaldehyde for histology or rinsed with saline, cleaned, and stored at −80°C for further analysis.

#### 2.3.4 Investigation of QGHJ’s effects on diet, weight, and water intake

Throughout the study, rats were closely monitored, with measurements of food and water consumption, as well as weight changes.

#### 2.3.5 Micro-CT analysis of bone mineral density and microstructure

Femoral samples were precisely positioned within the scanning area and underwent Micro-CT (SkyScan1276, Bruker, Germany) scanning at 80 KV voltage, 88 μA current, and 9 μm resolution. Image analysis software was used to calculate osteoporosis-related parameters within a defined region of interest (ROI), including bone mineral density (BMD), bone volume fraction (BV/TV), trabecular number (Tb.N), and trabecular separation (Tb.Sp). The quantitative data obtained were then used for statistical analysis.

#### 2.3.6 H&E and masson’s trichrome staining observations of femur tissues

Distal femur tissues underwent decalcification in 10% EDTA solution for 1 month. The decalcification fluid was replaced weekly, and tissue hardness was monitored throughout the process. After decalcification, femur sections (5 μm thick) were dewaxed, rehydrated, and stained with hematoxylin for 10 min and eosin for 5 min, followed by dehydration, clearing, and mounting. For Masson’s trichrome staining, samples were fixed in Bouin’s solution for 24 h, dehydrated in a graded ethanol series, cleared in xylene, and stained with Masson’s trichrome, concluding with dehydration and mounting. Microscopic examination was performed on all sections to document the staining effects on tissue structure.

#### 2.3.7 Biomechanical testing of femur maximum load

Muscles and soft tissues were removed from the femurs, which were then air-dried and secured onto a three-point bending jig with a 20 mm span. A universal mechanical testing machine applied compression at a rate of 1 mm/min until complete bone fracture, and the maximum load was recorded.

#### 2.3.8 Biochemical evaluation of renal and liver functions, alongside fasting glucose levels

An automatic biochemical analyzer was used to evaluate renal and hepatic function, as well as fasting blood glucose (FBG). Daily checks and calibrations were performed on the analyzer according to standard operating procedures to ensure optimal performance. Prepared samples were placed into different reaction pools of the analyzer, with specific reagents automatically added as needed for the assays. Following a series of automated reactions and detections, the analyzer directly provided numerical values for each parameter.

#### 2.3.9 ELISA quantification of bone turnover markers, fasting insulin, and glycated hemoglobin in serum

Tartrate-resistant acid phosphatase 5b (TRAP5b) (JL12318, Shanghai Jinglelai Biotechnology Co., Ltd., China) and procollagen type I N-terminal propeptide (PINP) (JL13510, Shanghai Jinglelai Biotechnology Co., Ltd., China), as well as fasting insulin (FINS) (JL10692, Shanghai Jinglelai Biotechnology Co., Ltd., China) and glycated hemoglobin (HbA1c) (JL21014, Shanghai Jinglelai Biotechnology Co., Ltd., China), were determined in rat sera. Serum samples and standards were prepared according to the manufacturer’s instructions. ELISA was performed by sequentially adding standards/samples, biotinylated antibodies, and enzyme conjugates to the plate wells. Following washing and color development, OD values were measured using an ELISA reader. Concentrations were calculated using standard curves. Inter-group differences were assessed using appropriate statistical analyses.

#### 2.3.10 Western blot analysis of bone regulatory proteins RUNX2 and PPARγ

Protein was extracted from bone tissue homogenates and quantified. Proteins were separated using 7% SDS-PAGE and transferred to PVDF membranes. The membranes were blocked with 5% non-fat milk and incubated with specific primary antibodies: Runt-related transcription factor 2 (TA5186, Abmart, China) (RUNX2, 1:1000) and Peroxisome proliferator-activated receptor gamma (PA4778, Abmart, China) (PPARγ, 1:1000), followed by incubation with anti-rabbit IgG (1:5000) for 2 h β-actin was used as the internal control. Protein bands were visualized using chemiluminescence (ECL) reagents and quantified using ImageJ 1.5.3 software.

#### 2.3.11 RT-qPCR analysis of bone regulatory genes *RUNX2* and *PPARγ*

Total RNA was extracted from rat bone tissues using Trizol reagent. RNA concentration and purity were determined before reverse transcription using Takara’s kits (Table 1). *GAPDH* served as the reference gene. RT-qPCR was performed using StepOnePlus system (4376600, USA) with the following protocol: initial denaturation at 95°C for 15 s, followed by annealing at 60°C and extension at 95°C for 15 s.

**TABLE 1:** Sequences of Runx2 and PPARγ.

Name	S/AS	Sequence
RUNX2	Forward	GCC​TTC​CTC​TGC​TGC​CAT​TAG​TC
	Reverse	TCA​TTG​AAC​TCC​ACC​GTG​CCT​TC
PPARγ	Forward	ACG​ATG​CTG​TCC​TCC​TTG​ATG​AAC
	Reverse	ATG​ATG​TCG​CAG​AAT​GGC​TTC​CTC

### 2.4 Pharmacological study of QGHJ on BMSCs under oxidative stress

#### 2.4.1 Experimental cells

Rat bone marrow mesenchymal stem cells (BMSCs) (Catalogue No: CP-R131) were procured from Wuhan Puno Sai Life Science Ltd.

#### 2.4.2 BMSCs culture

Primary BMSCs were cultured in an incubator at 37°C with 5% CO2. When cells reached 80%–90% confluence, they were passaged, and the medium was replaced as needed (every 3 days). Cells between passages two and 5 were used for subsequent experiments.

#### 2.4.3 Morphological observation

BMSCs suspensions were seeded into fresh culture media. After 24 h, the adhesion and growth of primary and third-passage cells were observed.

#### 2.4.4 Immunofluorescence characterization of BMSCs surface markers CD34 and CD44

BMSCs fixed with 4% paraformaldehyde, and permeabilized with 0.1% Triton X-100 for 10–15 min. Cells were then incubated for 1 h in PBST containing 5% BSA to block non-specific binding. Primary antibodies against CD34 and CD44 were added and incubated overnight at 4°C. After three to five washes with PBST (5 min each), fluorescence-labeled secondary antibodies were applied and incubated for 1 h at 37°C in the dark. Cell nuclei were stained with DAPI for 5–10 min, slides were mounted with anti-fade solution, and images were captured using a fluorescence microscope.

#### 2.4.5 CCK-8 assay to plot BMSCs growth curve

Third-generation BMSCs were seeded in a 96-well plate at densities of 5 × 103, 1 × 104, and 2 × 104 cells per well. Cell viability was evaluated using the CCK-8 assay at 24, 48, 72, 96, 120, 144, and 168 h post-seeding, and growth curves were generated.

#### 2.4.6 Grouping and treatment for BMSCs

This study investigated the effects of QGHJ on the viability, and apoptosis of BMSCs under oxidative stress conditions. Six groups were set up: a blank control group (NC) under standard conditions; a high-glucose group (HG) with 200 mmol/L glucose simulating oxidative stress; a positive control group treated with 0.1 mg/mL metformin (MET); and QGHJ intervention groups with low (QGHJ-L, 1 g/L), medium (QGHJ-M, 3 g/L), and high (QGHJ-H, 5 g/L) doses. Each group was treated for 24 h after establishing the oxidative stress model to evaluate the antioxidative effects.

The experiment explored the impact of QGHJ on osteogenic differentiation of BMSCs under oxidative stress. Built upon the existing group divisions, an additional osteogenic differentiation contingent (OD) was instituted. Subsequent to the 24-h treatment for antioxidative interrogation, the OD ensemble, along with the established sets, were commenced on osteogenic induction medium (PD-008–200, Wuhan Prunobio Life Technology Co., Ltd., China) concurrently. The medium was replenished every 3 days across the board to advance differentiation.

#### 2.4.7 CCK-8 evaluation of QGHJ’s effect on normal BMSCs

The cytotoxicity of various QGHJ (1, 3, 5, 7, 9, and 11 g/L) was assessed using the CCK-8 assay. Third-generation BMSCs were seeded in a 96-well plate at a density of 5 × 10^3^ cells per well. Once the cells reached 80% confluence, the medium was replaced with different QGHJ, along with blank and control groups. Cell survival rates were measured at 24, 48, and 72 h post-QGHJ treatment by determining the absorbance at 450 nm using CCK-8 reagent after 2 h of incubation in the dark.

#### 2.4.8 CCK-8 assessment of QGHJ’s influence on the viability of BMSCs under oxidative stress

The effects of different QGHJ (1, 3, 5 g/L) and MET on the proliferation of BMSCs under hyperglycemic conditions were evaluated using the CCK-8 method. The absorbance at 450 nm for each group was measured at 24, 48, and 72 h. The calculation of cell survival rate was performed using the same method as described above.

#### 2.4.9 Flow cytometric analysis of BMSCs apoptosis

Twenty-four hours after treatment, cells from each group were harvested for analysis. Apoptotic levels were determined using an Annexin V-FITC/PI staining kit, with reactions carried out in the dark. Flow cytometry was used to quantify apoptosis rates across the groups, and the assay was performed in triplicate for reproducibility.

#### 2.4.10 Alkaline phosphatase (ALP) staining

On the 14th day of osteogenic induction, cells were fixed with 4% paraformaldehyde solution for 30 min. ALP staining was performed according to the instructions provided with the chromogenic reagent kit. After the reaction, cells were washed three times with PBS buffer to remove excess dye before imaging under a phase-contrast microscope.

#### 2.4.11 Alizarin red s (ARS) staining

On day 21, cell mineralization and osteoid formation were assessed using Alizarin Red S staining. Post-induction, cells were fixed with 4% paraformaldehyde for 30 min. The staining reaction was conducted according to the ARS staining reagent manual to evaluate extracellular matrix mineralization and nodule formation. After stopping the reaction, cells were washed with deionized water to remove residual dye. Stained cells were then observed and imaged using an inverted microscope.

#### 2.4.12 Western blot analysis of bone regulatory proteins RUNX2 and PPARγ

Fourteen days after osteogenic induction, Western blot was performed to detect RUNX2 and PPARγ proteins in all groups, following the methodology outlined in section 2.3.10.

#### 2.4.13 RT-qPCR for *RUNX2* and *PPARγ* regulation in bone

The expression levels of *Runx2* and *PPARγ* were quantified on day 14 of osteogenic induction using the RT-qPCR protocol mentioned in section 2.3.11.

### 2.5 Investigation into how QGHJ alleviated oxidative stress in T2DOP through the SIRT1/NRF2/HO-1 pathway

#### 2.5.1 ELISA assessment of SOD levels in BMSCs groups

SOD concentrations in cell supernatants from each group were measured with prepared samples, following the steps described in section 2.3.9.

#### 2.5.2 Biochemical determination of oxidative stress markers SOD, MDA, and GSH-PX in rat serum

Reagents and specimens were prepared and aliquoted into ELISA plates along with corresponding detection mixtures. After appropriate reactions, optical densities were measured, and concentrations of SOD, malondialdehyde (MDA), and glutathione peroxidase (GSH-PX) were calculated based on standard curves, facilitating the evaluation of oxidative stress levels.

#### 2.5.3 Flow cytometric assessment of reactive ROS levels in BMSCs

After 24 h of experimental treatment, cells were thoroughly washed with sterile PBS to remove residual culture media. Under light-protected conditions, treated cells were stained with the ROS-sensitive DCFH-DA fluorescent probe in serum-free culture medium and incubated at 37°C for 20 min, allowing for selective ROS-mediated fluorescence signal production. Flow cytometry was subsequently used to measure fluorescence intensity in a specific channel, with higher intensities indicating elevated intracellular ROS levels.

#### 2.5.4 Immunofluorescence detection of oxidative stress proteins SIRT1, NRF2, and HO-1 in rats and BMSCs

Twenty-four hours post-treatment, cells were prepared for immunofluorescence detection following the steps outlined in section 2.4.4.

#### 2.5.5 Western blot analysis of oxidative stress-related proteins SIRT1, NRF2, and HO-1 in rats and BMSCs

Immunoblotting was performed for cells post-24 h treatment using specific antibody incubation concentrations: NRF2 (TA0639, Abmart, China) (1:1000), HO-1 (TN24541, Abmart, China) (1:1000), SIRT1 (TA0639, Abmart, China) (1:1000), similar to the method in section 2.3.10.

### 2.6 Statistical methods

Data was analyzed using GraphPad Prism v8.02 software, and results were expressed as mean ± standard deviation. One-way ANOVA was used to determine statistical significance with *P* < 0.05 considered significant. Data are presented as mean ± standard deviation. Compared to NC group, ^*^ *P* < 0.05, ^**^ *P* < 0.01, ^***^ *P* < 0.001; compared with the T2DOP/HG group, ^#^ *P* < 0.05, ^##^ *P* < 0.01, ^###^ *P* < 0.001. To prevent bias, the study was conducted using a double-blind design. The experimenters responsible for administering treatments and analyzing data were blinded to the group allocation. The blinding codes were only revealed after the completion of data analysis.

## 3 Results

### 3.1 UPLC-HRMS analysis of QGHJ

**FIGURE 2:**
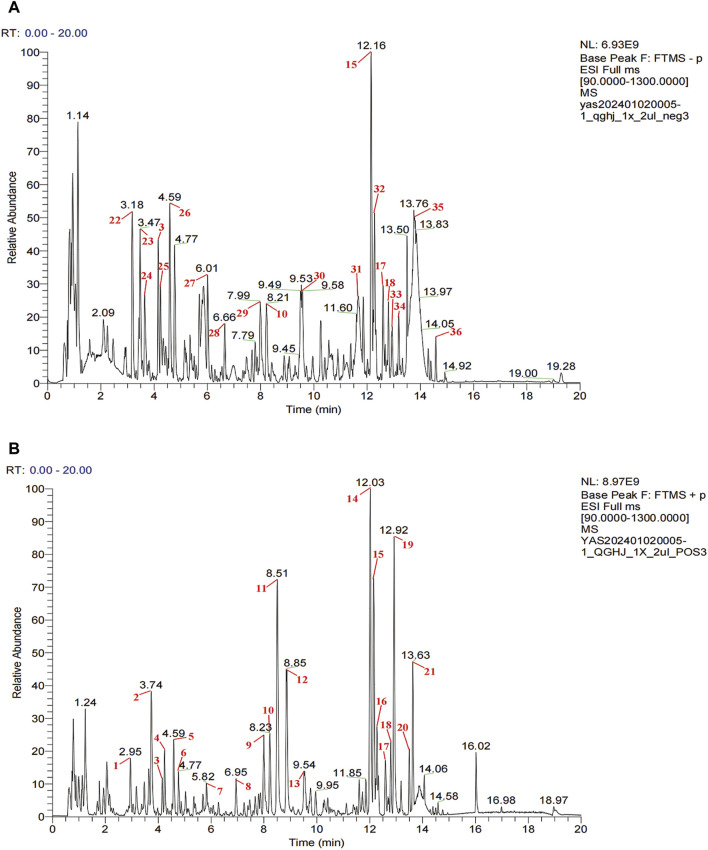
UPLC-HRMS analysis of QGHJ. **(A)** Positive ion mode of 40% methanol extract of QGHJ. **(B)** Negative ion mode of 40% methanol extract of QGHJ.

In QGHJ, ion base peak chromatograms and peak analysis confirmed 36 significant peaks in both ion modes. These were identified as primary compounds (Figure 2; Supplementary Table S1). Using the NPClassifier, the compounds were classified into superclasses, identifying six flavonoids, five isoflavones, five steroids, five monoterpenes, three coumarins, three cyclic polyketones, two phenylpropanoids, two triterpenes, and other individual compounds including one tryptophan alkaloid, one tyrosine alkaloid, a phenylpropanoid, and a fatty acid with its derivatives. Notably, E. brevicornu contributed flavonoids and the tyrosine alkaloid, P. corylifolia yielded isoflavones and coumarins, D. asperoides asper supplied monoterpenes and monoterpenoids, and L. chuanxiong was the primary source of cyclic polyketones.

### 3.2 QGHJ exhibited good safety in T2DOP rats

#### 3.2.1 QGHJ improved diet, body weight, and water intake

**FIGURE 3:**
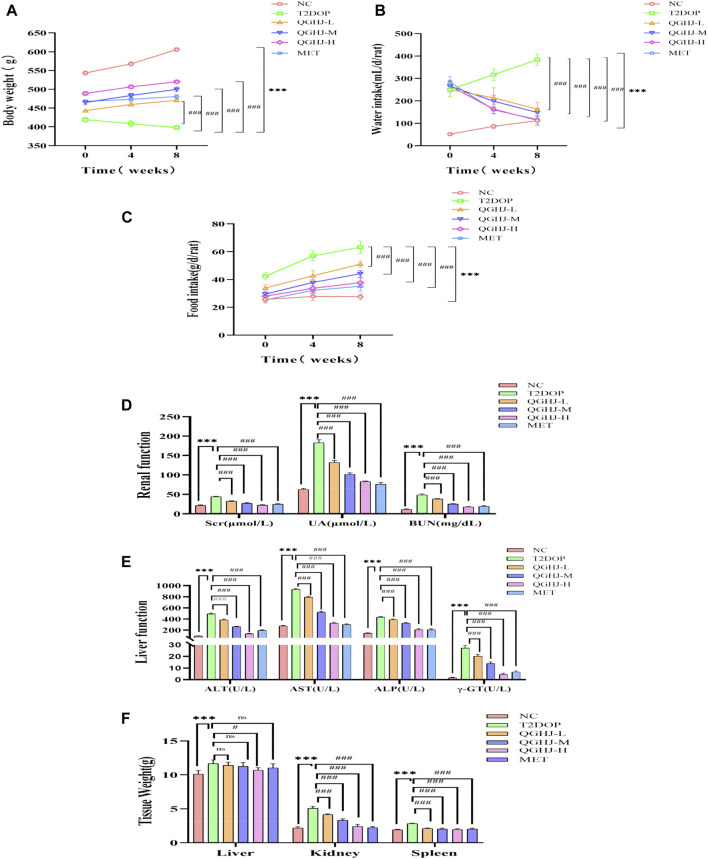
QGHJ demonstrated a favorable safety profile in T2DOP rats (n = 6). **(A–C)** Body weight, water intake, and food intake of different groups, respectively. Note: The *x*-axis indicated the timeline: 0 weeks (pre-treatment), 4 and 8 weeks (post-treatment). **(D, E)** Renal and liver function parameters. **(F)** Weights of the liver, kidneys, and spleen.

QGHJ increased body weight and reduced water and food intake in T2DOP rats. After 8 weeks of drug intervention, food and water intake significantly increased (T2DOP vs. NC, *P* < 0.001), while body weight decreased (T2DOP vs. NC, *P* < 0.001). QGHJ-L/M/H and MET exhibited significant decreases in food and water intake (QGHJ-L/M/H or MET vs. T2DOP, *P* < 0.001), and an increase in body weight (QGHJ-L/M/H or MET vs. T2DOP, *P* < 0.001) (Figures 3A–C). These results indicated that QGHJ can improve fundamental pathological features of T2DOP. Moreover, within the experimental dosage range, QGHJ did not exhibit any apparent toxicity in the T2DOP model.

#### 3.2.2 QGHJ enhanced renal and liver function

QGHJ significantly reduced renal function indicators such as serum creatinine (Scr), uric acid (UA), and blood urea nitrogen (BUN) in T2DOP rats. It also significantly reduced liver function markers such as alanine transaminase (ALT), aspartate transaminase (AST), alkaline phosphatase (ALP), and gamma-glutamyl transpeptidase (GGT). After 8 weeks, indicators of liver and renal functions significantly increased (T2DOP vs. NC, *P* < 0.001). Markedly reduced markers of renal and liver function were observed (QGHJ-L/M/H or MET vs. T2DOP, *P* < 0.001) (Figures 3D, E). The results suggested that during the experiment, QGHJ did not cause noticeable toxic damage to the liver and kidneys of rats; on the contrary, it improved liver and kidney function abnormalities caused by diabetes, further supporting the safety and efficacy of QGHJ in treating this disease.

#### 3.2.3 QGHJ ameliorated liver, spleen, and kidney weights

QGHJ was observed to ameliorate the weights of the liver, spleen, and kidneys in T2DOP rats. Following 8 weeks of treatment, significant increases in the weights of the liver, spleen, and kidneys were noted (T2DOP vs. NC, *P* < 0.001). Compared to T2DOP rats, QGHJ-L, QGHJ-M, and MET administrations did not significantly improve liver weight. However, the QGHJ-H significantly reduced liver weight (QGHJ-H vs. T2DOP, *P* < 0.001). All QGHJ treatments and MET significantly decreased the weights of the spleen and kidneys (QGHJ or MET vs. T2DOP, *P* < 0.001) (Figure 3F).

### 3.3 QGHJ exerted a bone-protective effect in T2DOP rats

#### 3.3.1 QGHJ attenuated bone mass loss and regulated biomechanical parameters of femurs

**FIGURE 4:**
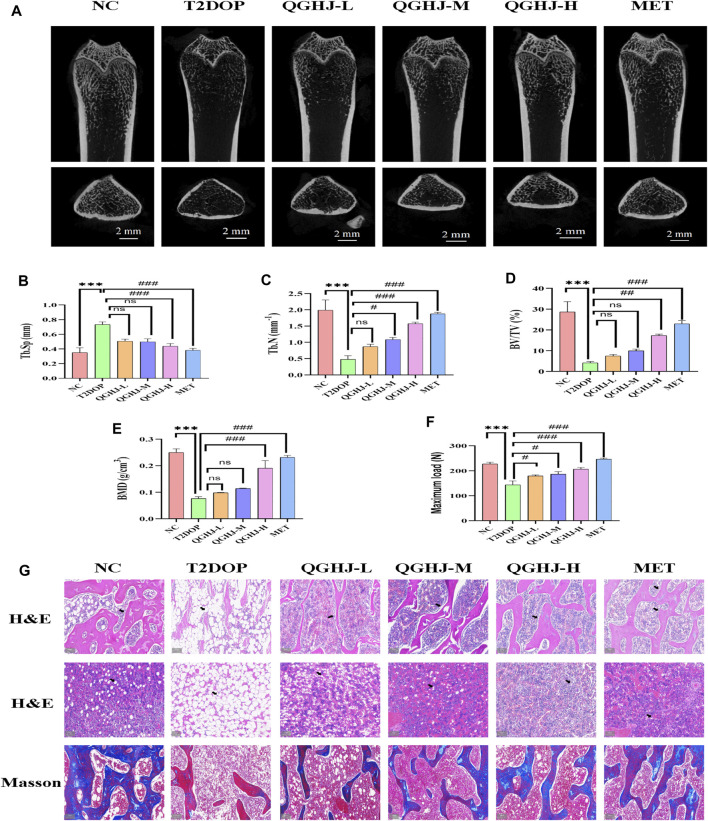
QGHJ exerted a bone-protective effect in T2DOP rats (n = 3). **(A)** Representative micro-CT images of distal femurs (scale bar = 2 mm). **(B–E)** Quantitative analysis of trabecular parameters: Tb. Sp, Tb.N, BV/TV, and BMD. **(F)** Maximum load. **(G)** H&E staining of distal femurs and bone marrow cavities. Black arrows indicated trabeculae and adipocyte in bone marrow cavities. Masson’s trichrome staining showing osteoid formation (blue areas) and mature, mineralized bone tissue (red areas) (scale bar = 200 μm).

In the T2DOP group, micro-CT scans revealed sparse trabecular structures and significant bone mass loss in the femurs. Quantitative analysis showed notably decreased BMD, BV/TV, and Tb.N (T2DOP vs. NC, *P* < 0.001), and substantially increased Tb. Sp (T2DOP vs. NC, *P* < 0.001). After 8 weeks of treatment, QGHJ-H and MET markedly improved BMD and BV/TV (QGHJ-H or MET vs. T2DOP, *P* < 0.001), while QGHJ-M/H and MET significantly increased Tb.N (QGHJ-M/H, or MET vs. T2DOP, *P* < 0.001) and decreased Tb. Sp (QGHJ-H or MET vs. T2DOP, *P* < 0.001) (Figures 4A–E). Biomechanical properties assessed by three-point bending tests demonstrated a significant reduction in the maximum load borne by T2DOP rat femurs (T2DOP vs. NC, *P* < 0.001). QGHJ-L/M significantly increased the maximum load (QGHJ-L/M vs. T2DOP, *P* < 0.05), with QGHJ-H and MET further enhancing it (QGHJ-H or MET vs. T2DOP, *P* < 0.001) (Figure 4F).

#### 3.3.2 QGHJ improved bone microstructure and inhibited marrow adipogenesis

Femoral trabecular structure, new bone formation, and marrow adipogenesis were evaluated in T2DOP rats treated with QGHJ or MET. In the NC group, femoral trabeculae exhibited a dense, moderately thick, and well-ordered distribution, accompanied by abundant new and mature bone tissue, while the bone marrow adipocytes were sparsely distributed with low cell density. Conversely, T2DOP rats exhibited trabecular depletion, thinning, and disorganization, along with reduced new bone formation and increased marrow adipocyte density and volume. QGHJ and MET treatments improved trabecular structure, promoted new bone formation, and inhibited marrow adipogenesis to varying degrees in a dose-dependent manner. The most significant improvements were observed in the QGHJ-M/H and MET groups, while the QGHJ-L group showed limited efficacy (Figure 4G).

#### 3.3.3 QGHJ stabilized serum bone turnover biomarkers TRAP5b and PINP

**FIGURE 5:**
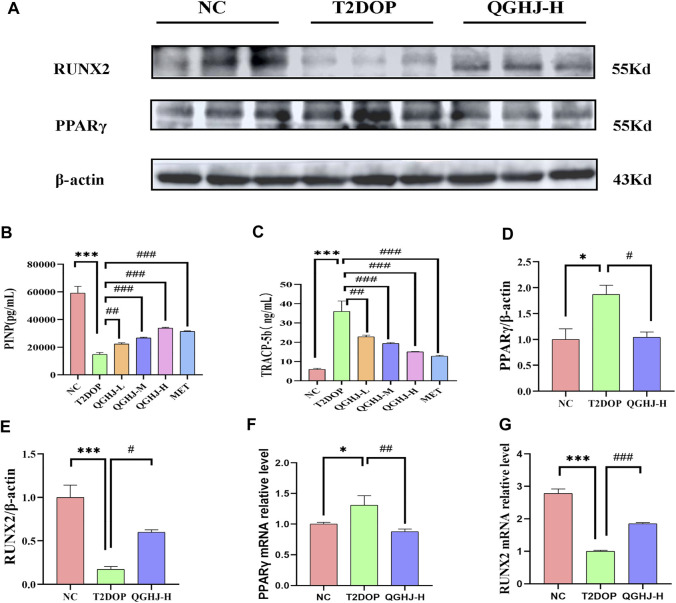
Effects of QGHJ on bone biomarkers in T2DOP rats. **(A)** Western blot analysis of RUNX2 and PPARγ protein expression. β-actin served as the internal control (n = 3). **(B, C)** Serum levels of TRAP5b and PINP across groups (n = 6). **(D, E)** Relative protein expression of RUNX2 and PPARγ, normalized to β-actin (n = 3). **(F, G)** *RUNX2* and *PPARγ* mRNA expression levels in bone tissues, n = 3.

The ELISA results demonstrated a significant elevation in TRAP5b and a concomitant decrease in PINP (T2DOP vs. NC, *P* < 0.001). After 8 weeks of treatment, the QGHJ-L group showed a significant elevation in TRAP5b (QGHJ-L vs. T2DOP, *P* < 0.01) and a marked decrease in PINP (QGHJ-L vs. T2DOP, *P* < 0.01). The QGHJ-M/H, and MET groups demonstrated a significant increase in TRAP5b (QGHJ-M/H or MET vs. T2DOP, *P* < 0.001) and a significant decrease in PINP (QGHJ-M/H or MET vs. T2DOP, *P* < 0.001) (Figures 5B, C).

#### 3.3.4 QGHJ modulated bone regulatory proteins RUNX2 and PPARγ

Western blot analysis of femur samples assessed RUNX2 and PPARγ expression. The expression level of RUNX2 protein was found to be decreased (T2DOP vs. NC, *P* < 0.001), whereas PPARγ protein expression was elevated (T2DOP vs. NC, *P* < 0.001). After 8 weeks of treatment, QGHJ-H significantly increased RUNX2 protein expression (QGHJ-H vs. T2DOP, *P* < 0.001) and significantly decreased PPARγ protein levels (QGHJ-H vs. T2DOP, *P* < 0.001) (Figures 5A, D, E).

#### 3.3.5 QGHJ improved bone regulatory genes *RUNX2* and *PPARγ*

Expression levels of *RUNX2* mRNA in femur tissues were reduced (T2DOP vs. NC, *P* < 0.001), while *PPARγ* mRNA levels were increased (T2DOP vs. NC, *P* < 0.001). Treatment with QGHJ-H for 8 weeks led to a significant increase in *RUNX2* mRNA expression (QGHJ-H vs. T2DOP, *P* < 0.001) and a significant decrease in *PPARγ* mRNA levels (QGHJ-H vs. T2DOP, *P* < 0.001) (Figures 5F, G).

### 3.4 QGHJ stabilized glycemic metabolism

**FIGURE 6:**
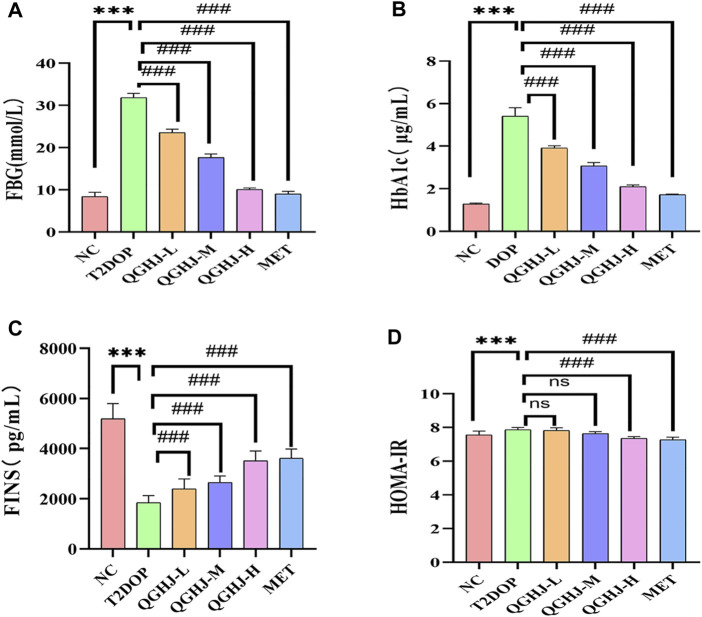
Effects of QGHJ on glycemic metabolism in T2DOP rats (n = 6). **(A–D)** Levels of FBG, HbA1c, FINS, and HOMA-IR across different groups.

Biochemical and ELISA analyses revealed that HbA1c, FBG, and the insulin resistance index (HOMA-IR) were significantly elevated, while FINS levels were markedly reduced (T2DOP vs. NC, *P* < 0.001). After 8 weeks of treatment, QGHJ-L/M/H and MET significantly lowered HbA1c and FBG levels (QGHJ-L/M/H or MET vs. T2DOP, *P* < 0.001). FINS notably increased with QGHJ-L/M/H or MET treatment (QGHJ-L/M/H, or MET vs. T2DOP, *P* < 0.001). No statistical significance was found for HOMA-IR in the QGHJ-L/M groups. However, HOMA-IR significantly decreased in the QGHJ-H and MET groups (QGHJ-H or MET vs. T2DOP, *P* < 0.001) (Figure 6).

### 3.5 Identification of BMSCs

#### 3.5.1 Morphological observation of BMSCs

**FIGURE 7:**
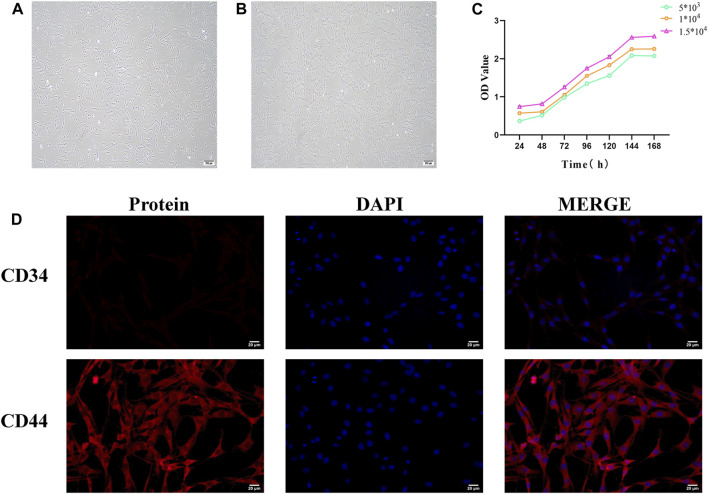
Identification of bone marrow mesenchymal stem cells (BMSCs). **(A)** Morphology of primary cultured BMSCs (scale bar = 200 μm). **(B)** Morphology of third-generation BMSCs (scale bar = 200 μm). **(C)** BMSC growth curve. **(D)** Immunofluorescence staining of BMSC surface markers (scale bar = 20 μm).

Inverted microscopy revealed that 24 h after primary culture, rat BMSCs began to adhere and grow, with a small number of spindle-shaped cells visible. After three passages, the obtained BMSCs had a purity exceeding 95%, exhibiting a spindle-shaped morphology and a swirling growth pattern (Figures 7A, B).

#### 3.5.2 Plotting the growth curve of BMSCs

The CCK8 assay results showed that the growth curve of BMSCs exhibited a flat “S” shape trend, including a latent phase (days 1–2), a logarithmic growth phase (days 3–6), and a plateau phase (after day 6). At different seeding densities, the growth curves of BMSCs displayed similar three growth stages: initial adaptation delay, rapid proliferation, and stable growth. The experimental data reflected the typical growth pattern of BMSCs. Different seeding densities did not significantly affect the overall shape of the growth curve but influenced the specific time points at which cells entered the logarithmic and plateau phases (Figure 7C).

#### 3.5.3 Detection of BMSCs surface markers

CD44 and CD34 are specific surface markers for BMSCs. CD44 is a positive marker, while CD34 is a negative surface marker. Immunofluorescence analysis demonstrated that the expression level of CD44-positive cells was extremely high in purified BMSCs, whereas the proportion of CD34-positive cells was remarkably low. This confirms that the vast majority of BMSCs express CD44 but not CD34 molecules (Figure 7D).

### 3.6 Assessment of BMSCs viability

#### 3.6.1 Effects of QGHJ on normal BMSCs

**FIGURE 8:**
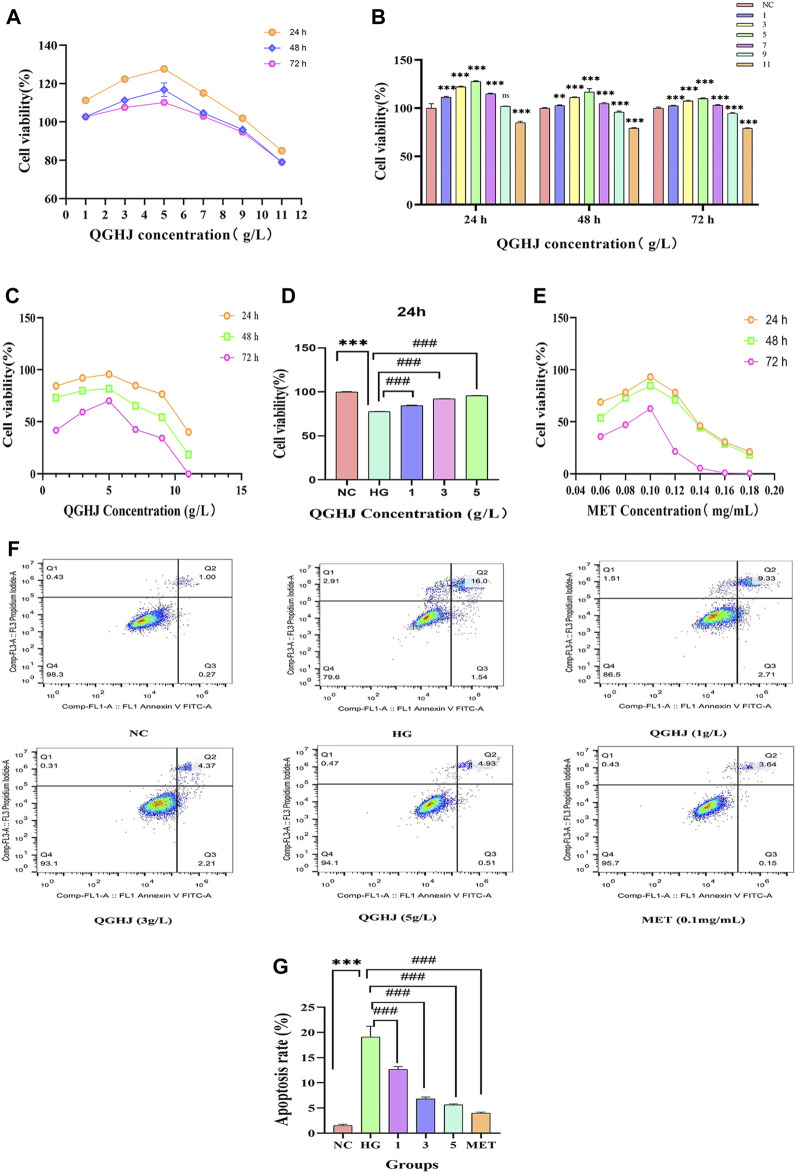
Effects of QGHJ on BMSC viability and apoptosis. **(A, B)** QGHJ effects on normal BMSC viability (n = 9). **(C, D)** QGHJ effects on BMSC proliferation under high glucose conditions (n = 9). **(E)** Effects of MET (0.1 mg/mL) on BMSC growth in hyperglycemic conditions (n = 9). **(F, G)** QGHJ effects on BMSC apoptosis (n = 3).

The results indicated that at the 24-h time point, various concentrations of QGHJ had little impact on the cell viability of BMSCs, showing no apparent cytotoxicity compared to the normal control group. However, at 48 and 72 h, high concentrations of QGHJ (≥5 g/L) exhibited a certain degree of cytotoxicity, with cell viability gradually decreasing as the concentration increased (Figures 8A, B).

#### 3.6.2 QGHJ improved the viability of BMSCs under oxidative stress

At the 24-h, 36-h, and 48-h time points, QGHJ at three concentration gradients of 1 g/L, 3 g/L, and 5 g/L significantly enhanced the cell proliferation activity of BMSCs under oxidative stress induced by high glucose. Among them, 5 g/L QGHJ-H group exhibited the most remarkable promoting effect at 24 h, with cell proliferation activity approaching the normal control level (Figure 8C). Therefore, 5 g/L was preliminarily determined as the optimal QGHJ concentration for promoting BMSCs proliferation. As shown in Figure 8D, at the 24-h time point, all concentrations of QGHJ significantly reversed the proliferation inhibition induced by high glucose (QGHJ vs. HG, *P* < 0.001). Additionally, at 24 h, 0.1 mg/mL was determined to be the optimal MET concentration for promoting BMSC proliferation (Ren et al., 2021) (Figure 8E).

### 3.7 QGHJ ameliorated apoptosis of BMSCs under oxidative stress

Initially, we employed flow cytometry to assess the apoptosis levels of BMSCs in each group. The results demonstrated that the apoptosis rate of cells in the HG group was significantly elevated (HG vs. NC, *P* < 0.001), suggesting that oxidative stress induced by high glucose induces excessive apoptosis of BMSCs, leading to decreased cell viability. Subsequently, we treated HG group cells with different concentrations of QGHJ and MET. The QGHJ groups and MET significantly reduced apoptosis (QGHJ-L/M/H, or MET vs. HG, *P* < 0.001) (Figures 8F, G). These findings preliminarily confirm that QGHJ possesses clear anti-apoptotic activity and can effectively counteract the excessive apoptosis of BMSCs caused by oxidative stress, thereby maintaining cell survival.

### 3.8 QGHJ mitigated oxidative stress-induced inhibition of osteogenic differentiation in BMSCs

#### 3.8.1 ALP staining

**FIGURE 9:**
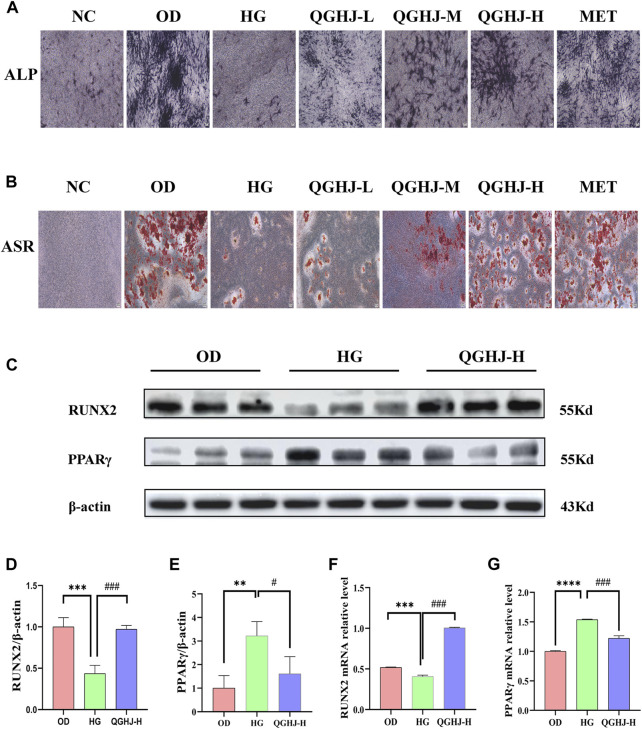
Effects of QGHJ on oxidative stress-induced inhibition of BMSC osteogenic differentiation (n = 3). **(A)** Representative ALP staining images (scale bar = 200 μm). **(B)** Representative ASR staining images (scale bar = 200 μm). **(C–E)** Western blot analysis of RUNX2 and PPARγ in BMSCs. **(F, G)** RT-qPCR analysis of *RUNX2* and *PPARγ* mRNA levels.

ALP staining was primarily performed to detect the early osteogenic differentiation ability of cells. On day 14 of osteogenic differentiation, the ALP staining intensity of the OD group was significantly enhanced compared to the NC group, indicating that we successfully induced the early osteogenic differentiation of BMSCs. However, the ALP staining was weakened in the HG group compared to the OD group, suggesting that oxidative stress induced by high glucose has an inhibitory effect on early bone formation. Notably, the ALP staining intensity was significantly enhanced in all treatment groups compared to the HG group. These results suggest that QGHJ can significantly improve the early osteogenic differentiation ability of BMSCs under oxidative stress, partially reversing the osteogenic differentiation inhibition (Figure 9A).

#### 3.8.2 ASR staining

On day 21 of osteogenic differentiation, we performed SR staining to examine the extracellular matrix calcification in each group. The results showed that the alizarin red staining was more pronounced in the OD group compared to the NC group. The staining was significantly weakened in the HG group compared to the OD group. Compared to the HG group, all treatment groups formed more calcified nodules with more prominent alizarin red staining. This indicates that QGHJ can effectively promote the late osteogenic differentiation of BMSCs under oxidative stress induced by high glucose, enhancing the extracellular matrix calcification and bone tissue formation ability (Figure 9B).

#### 3.8.3 QGHJ regulated the bone regulatory proteins RUNX2 and PPARγ in BMSCs

Western blot was used to detect RUNX2 and PPARγ in the OD, HG, and QGHJ-H groups after 14 days of osteogenic induction. The expression level of RUNX2 was decreased (HG vs. NC, *P* < 0.001), while the protein expression level of PPARγ was increased (HG vs. NC, *P* < 0.01). After treatment, QGHJ-H significantly increased the protein expression of RUNX2 (QGHJ-H vs. HG, *P* < 0.001), and the protein expression level of PPARγ was significantly decreased (QGHJ-H vs. HG, *P* < 0.05) (Figures 9C–E).

#### 3.8.4 QGHJ regulated the bone regulatory genes *RUNX2* and *PPARγ* in BMSCs

RT-qPCR detected that the mRNA expression level of *RUNX2* was decreased (HG vs. NC, *P* < 0.001), while the mRNA expression level of *PPARγ* was increased (HG vs. NC, *P* < 0.001). After treatment, QGHJ-H significantly increased the mRNA expression of RUNX2 (QGHJ-H vs. HG, *P* < 0.001), and the mRNA expression level of *PPARγ* was significantly decreased (QGHJ-H vs. HG, *P* < 0.001) (Figures 9F, G).

### 3.9 The mechanism of QGHJ activating the SIRT1/NRF2/HO-1 pathway to reduce oxidative stress in T2DOP

#### 3.9.1 QGHJ reduced oxidative stress indicators MDA, SOD, and GSH-PX

**FIGURE 10:**
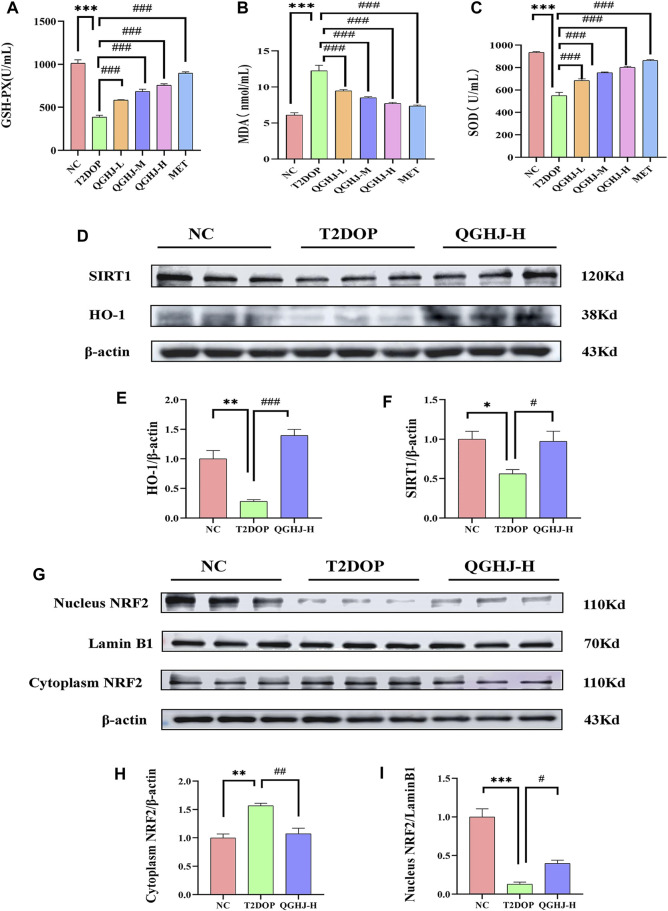
Serum oxidative stress levels and protein expression levels of SIRT1/NRF2/HO-1 in the femur. **(A–C)** GSH-PX activity, MDA content, and SOD activity in each group (n = 6). **(D–F)** Relative protein expression levels of SIRT1 and HO-1 quantified by grayscale analysis and normalized to β-actin. **(G, I)** Relative nuclear NRF2 protein expression quantified by grayscale analysis and normalized to Lamin B1. **(G, H)** Relative cytoplasmic NRF2 protein expression quantified by grayscale analysis and normalized to β-actin.

Biochemical test results showed that the levels of GSH-PX and SOD in the serum of the T2DOP group were significantly decreased, while the MDA level was significantly increased (T2DOP vs. NC, *P* < 0.001), reflecting a notable oxidative stress state in the T2DOP group. After 8 weeks of treatment, the levels of GSH-PX and SOD were significantly increased, and the MDA level was significantly decreased in the QGHJ-L/M/H, and MET groups (QGHJ-L/M/H, or MET vs. T2DOP, *P* < 0.001). This suggested that QGHJ and MET could effectively improve the oxidative stress condition in T2DOP rats (Figures 10A–C).

#### 3.9.2 Immunofluorescence detection of SIRT1/NRF2/HO-1 in femur

**FIGURE 11:**
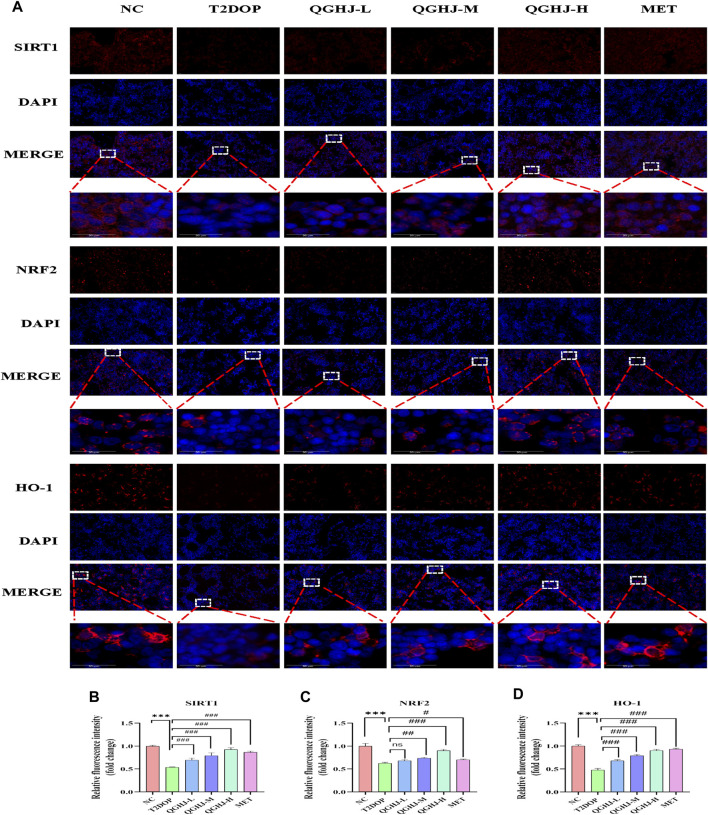
Immunofluorescence analysis of SIRT1, NRF2, and HO-1 in femur (n = 3). **(A)** Representative image (scale bar = 20 μm). Note: White boxes in NRF2 images indicated nuclear translocation. **(B–D)** Quantitative analyses of SIRT1, NRF2, and HO-1 expression.

The immunofluorescence results of animal bone tissue showed that the protein expression levels of SIRT1, NRF2, and its downstream effector molecule HO-1, as well as the nuclear translocation level of NRF2, were decreased in the T2DOP group (T2DOP vs. NC, *P* < 0.001), suggesting that the SIRT1/NRF2/HO-1 antioxidant pathway was inhibited in the T2DOP group. After 8 weeks of intervention with QGHJ or MET, the protein expression levels of SIRT1, NRF2, and HO-1 gradually increased (QGHJ-M/H, or MET vs. T2DOP, *P* < 0.001), and the nuclear translocation level of NRF2 also increased in these treatment groups. These results suggest that QGHJ can upregulate the activity of the SIRT1/NRF2/HO-1 antioxidant pathway, promote the nuclear translocation of NRF2, and activate the expression of downstream antioxidant genes, thereby exerting antioxidant protective effects (Figure 11).

#### 3.9.3 Western blot detection of SIRT1/NRF2/HO-1 in femur

Western blot detection showed that the expression levels of SIRT1 and HO-1 in the total protein of the femur were significantly decreased (T2DOP vs. NC, *P* < 0.05 and *P* < 0.01), while SIRT1 was significantly increased in the QGHJ-H (QGHJ-H vs. T2DOP, *P* < 0.05), and HO-1 was significantly increased (QGHJ-H vs. T2DOP, *P* < 0.001). The nuclear protein level of NRF2 was decreased (T2DOP vs. NC, *P* < 0.001), but the cytoplasmic protein level was increased (T2DOP vs. NC, *P* < 0.01), suggesting that NRF2 underwent nuclear-to-cytoplasmic translocation, indicating that the antioxidant mechanism was inhibited in the T2DOP group. In contrast, the nuclear protein level of NRF2 was increased (QGHJ-H vs. T2DOP, *P* < 0.05), and the cytoplasmic protein level was decreased (QGHJ-H vs. T2DOP, *P* < 0.01), suggesting that QGHJ-H may exert its effects by promoting the nuclear translocation of NRF2 from the cytoplasm. These results indicate that QGHJ-H may exert antioxidant effects by upregulating SIRT1 and promoting the nuclear translocation of NRF2 to activate HO-1 (Figures 10D–I).

#### 3.9.4 QGHJ reduced oxidative stress indicators SOD and ROS in BMSCs

**FIGURE 12:**
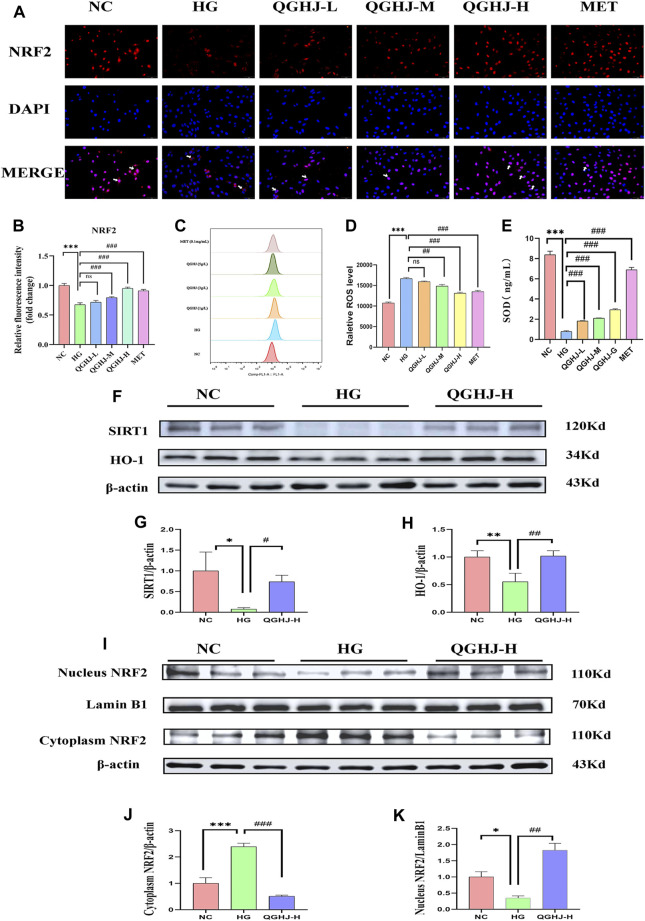
Analysis of NRF2 pathway activation and oxidative stress markers in BMSCs (n = 3). **(A)** Representative immunofluorescence images showing NRF2 expression and nuclear translocation in BMSCs. White arrows indicated NRF2 nuclear translocation. **(B)** Quantification of NRF2 immunofluorescence intensity. **(C, D)** Intracellular ROS levels in different treatment groups. **(E)** SOD activity in cells from each experimental group. **(F–K)** Western blot analysis quantified the protein expression levels of SIRT1, NRF2, and HO-1 in BMSCs. Expression levels of SIRT1 and HO-1 were normalized against β-actin, while nuclear NRF2 expression was compared to Lamin B1 and cytoplasmic NRF2 expression to β-actin.

Flow cytometry results of intracellular ROS levels showed that the ROS content in BMSCs of the HG group was significantly increased (HG vs. NC, *P* < 0.001). After intervention, the intracellular ROS levels were significantly reduced (QGHJ-M/H, or MET vs. HG, *P* < 0.01). This indicated that QGHJ and MET can effectively clear the excessive ROS induced by high glucose in BMSCs, thereby improving the cellular oxidative stress condition (Figures 12C, D).

ELISA detection showed that the SOD activity in BMSCs was significantly decreased (HG vs. NC, *P* < 0.001), reflecting an oxidative stress state in the HG group cells. After intervention with QGHJ or MET, the cellular SOD activity was significantly increased (QGHJ-L/M/H or MET vs. HG, *P* < 0.001), respectively, suggesting that these two groups could effectively alleviate oxidative stress (Figure 12E).

#### 3.9.5 Immunofluorescence detection of NRF2 in BMSCs

The cellular immunofluorescence results showed that under HG-induced oxidative stress conditions, the expression and nuclear translocation levels of NRF2 were significantly decreased (HG vs. NC, *P* < 0.001), suggesting that the NRF2-mediated antioxidant stress response was inhibited. After 24 h of intervention with QGHJ or MET, the expression and nuclear translocation levels of NRF2 were significantly increased in the QGHJ-M/H, and MET groups (QGHJ-M/H, or MET vs. HG, *P* < 0.001), while no significant changes were observed in the QGHJ-L group. These results indicated that QGHJ and MET could effectively reverse the HG-induced decrease in NRF2 expression and inhibition of nuclear translocation, thereby restoring the NRF2-mediated cellular antioxidant stress capacity. Notably, the effect of QGHJ on NRF2 exhibited a dose-dependent relationship at different doses (Figures 12A, B).

#### 3.9.6 Western blot detection of SIRT1/NRF2/HO-1 in BMSCs

Western blot results showed that in the BMSCs of the HG group, the protein expression of SIRT1 and HO-1 was significantly downregulated (HG vs. NC, *P* < 0.05 and *P* < 0.01), and NRF2 underwent nuclear protein downregulation (HG vs. NC, *P* < 0.05) and cytoplasmic protein upregulation (HG vs. NC, *P* < 0.001), indicating its translocation from the nucleus to the cytoplasm and suggesting impaired antioxidant capacity. In contrast, SIRT1 expression was significantly upregulated (QGHJ-H vs. HG, *P* < 0.05), HO-1 expression was also significantly upregulated (QGHJ-H vs. HG, *P* < 0.01), and NRF2 underwent nuclear protein upregulation (QGHJ-H vs. HG, *P* < 0.01) and cytoplasmic protein downregulation (QGHJ-H vs. HG, *P* < 0.001), translocating from the cytoplasm to the nucleus, indicating enhanced antioxidant function. These results were consistent with the Western blot results of bone tissue, suggesting that QGHJ-H may exert antioxidant stress effects through the SIRT1/NRF2/HO-1 pathway, thereby protecting the skeletal system (Figures 12F–K).

## 4 Discussion

Our research findings revealed that QGHJ demonstrated safety in vivo experiments, exerting protective effects on bone and regulating blood glucose levels. *In vitro* experiments showed that QGHJ enhanced BMSC viability and osteogenic differentiation under high glucose conditions. Both *in vivo* and *in vitro* experiments demonstrated that QGHJ activated the SIRT1/NRF2/HO-1 pathway and promoted NRF2 nuclear translocation, suppressing oxidative stress. Using UPLC-HRMS, we identified the major active components of QGHJ, including flavonoids, isoflavones, steroids, and monoterpenes. These components exhibited synergistic effects in bone protection, glucose metabolism improvement, and antioxidant properties.

Our research findings revealed that QGHJ regulated bone metabolism-related factors, such as PINP, TRACP 5b, RUNX2, and PPARγ, to maintain bone homeostasis. Studies on bone metabolism regulatory factors have provided crucial evidence for diagnosing and treating bone metabolism-related diseases. Bone turnover biomarkers PINP and TRACP 5b reflect bone formation and resorption status, respectively. Monitoring their dynamic changes helps assess alterations in bone quality (Eastell and Szulc, 2017). Type I collagen, the predominant bone matrix protein in the skeleton, is a crucial component of the organic matrix (Koivula et al., 2012). During active type I collagen synthesis, osteoblasts cleave the precursor protein and release PINP, with its concentration level directly reflecting osteoblast activity within the bone tissue. TRACP 5b is recognized as a biomarker for evaluating bone resorption status. During bone mineral dissolution, osteoclasts secrete various acidic hydrolases, such as TRACP 5b, which participate in bone matrix degradation (Lv et al., 2015). RUNX2, a bone-specific transcription factor, plays a vital role in osteoblast differentiation and bone formation (Komori, 2022). RUNX2 gene knockout mice exhibit reduced BMD, decreased trabecular bone volume, and lower levels of bone formation and resorption markers (Qin et al., 2021). Research has shown that elevated ROS levels lead to RUNX2 oxidation and proteasomal degradation, inhibiting osteoblast differentiation (Hu et al., 2023). PPARγ, another critical regulatory factor, maintains the multi-directional differentiation potential of the osteoblast lineage when expressed at moderate levels. However, PPARγ overactivation promotes adipogenic differentiation and suppresses osteogenic differentiation, suggesting its potential as a therapeutic target for bone metabolism (Liu C. et al., 2022).

The role of QGHJ in maintaining bone homeostasis can be attributed to the synergistic bone-protective effects of its components, such as icariin, psoralidin, corylin, and 17α-estradiol. Icariin, a flavonoid, modulates miRNA expression, enhancing cell viability, inhibiting apoptosis, and promoting migration and angiogenesis (Zhang Q. et al., 2022). Psoralidin, an isoflavone, promotes calcium nodule formation, ALP activity, and osteocalcin levels in MC3T3-E1 cells, suggesting its potential in bone formation promotion and osteoporosis treatment (Cao et al., 2019). Corylin activates the Wnt/β-catenin and estrogen receptor pathways, thereby enhancing osteoblast differentiation and mineralization while upregulating RUNX2 and ALP expression (Yu et al., 2020). 17α-estradiol, a steroid, inhibits weight gain and obesity in ovariectomized mice while maintaining uterine weight, endometrial morphology, and improving BMD (Mann et al., 2020). Loganin, a monoterpene, promotes mouse osteoblast precursor differentiation into mature osteoblasts by regulating differentiation marker mRNA expression, thus improving BMD and bone microstructure (Lee et al., 2022). Sweroside increases bone mineral content and density while reducing bone marrow adipocyte formation. It also enhances ALP activity and upregulates RUNX2 expression (Choi et al., 2021). Loganic acid promotes osteoblast differentiation, enhances ALP activity, prevents BMD loss in ovariectomized mice, and improves bone structure (Park et al., 2020b). Ligustilide ameliorates prednisone-induced inhibition of bone formation in zebrafish by promoting osteoblast differentiation and survival, inhibiting apoptosis, and activating the EGFR and ERK1/2 pathways (Yang et al., 2019). Asperosaponin VI improves bone volume fraction, suppresses the reduction of trabecular number and separation of cancellous trabeculae, and maintains the structural model index (Niu et al., 2023). Additionally, bavachinin and cholic acid, components of QGHJ, may synergistically influence glucose metabolism. Bavachinin and protodioscin, flavonoids that act as selective modulators of PPARγ, increase the osteogenic activity of MC3T3-E1 cells, inhibit RANKL-induced differentiation of RAW264.7 cells, improve insulin sensitivity, and maintain bone mass and biomechanical properties (Liu et al., 2023). Cholic acid, a steroid, improves glucose tolerance, reduces total fat and energy efficiency, and decreases fasting blood glucose and insulin resistance (Ippagunta et al., 2018).

Our study revealed that QGHJ activated the SIRT1/NRF2/HO-1 pathway and facilitated NRF2 nuclear translocation. This effect may be attributed to the synergistic antioxidant properties of QGHJ components, including Psoralidin, 17β-estradiol, Sweroside, Loganic acid, and Ligustilide. Psoralidin, an isoflavone, suppresses COX-2 and ROS production in rat osteoblasts, thereby protecting bone tissue (Zhai et al., 2017) and activating the SIRT1/NRF2/HO-1 axis (Lei et al., 2022). 17β-estradiol, a steroid, binds to estrogen receptors and promotes SIRT1 deacetylation, activating the SIRT1/NRF2/HO-1 pathway in BMSCs (Tian et al., 2023; Yang et al., 2023). Loganic acid, a monoterpene, modulates the SIRT1/NRF2 pathway. Activated SIRT1 translocates to the nucleus through deacetylation, further activating NRF2 and inhibiting oxidative stress and inflammation (Prakash et al., 2023). Ligustilide is a known novel SIRT1 agonist that binds to SIRT1 and enhances its deacetylation activity (Li G. et al., 2023). The SIRT1/NRF2/HO-1 signaling pathway is a promising therapeutic target for oxidative stress-related diseases (Mukai et al., 2022) and plays a pivotal role in regulating skeletal homeostasis (Kubo et al., 2019). Studies have demonstrated that resveratrol activates the SIRT1/NRF2 pathway, mitigating oxidative stress and improving osteogenic potential in human dental pulp stromal cells (Zhang J. et al., 2022). Moreover, additional studies have revealed that the SIRT1/NRF2/HO-1 pathway promotes BMSC osteogenic differentiation by augmenting RUNX2 transactivation (Zainabadi et al., 2017; Mccarty et al., 2022). SIRT1, a member of the Sirtuin family, is a nicotinamide adenine dinucleotide-dependent deacetylase involved in oxidative stress processes (Cui et al., 2022). Upon activation, SIRT1 undergoes deacetylation and translocates to the nucleus, where it activates downstream transcription factors NRF2 and HO-1. These factors are crucial for bone metabolism and antioxidant stress response (Sun et al., 2018). In MC3T3-E1 cells, SIRT1 inhibitor (EX527) and Nrf2 inhibitor (ML385) have been shown to downregulate the expression of osteogenesis-related markers RUNX2 and ALP (Zhang et al., 2024). Similarly, another study has demonstrated that SIRT1 inhibitor EX-527 suppresses both SIRT1 expression and Nrf2 protein levels, indicating that NRF2 is a crucial downstream target in the SIRT1 signaling pathway (Kou et al., 2023). NRF2, a key transcription factor regulating antioxidant defense, is a potential therapeutic target for oxidative stress-related diseases. Under oxidative stress conditions, NRF2 remains inactive when bound to Kelch-like ECH-associated protein 1 (KEAP1). QGHJ treatment activates SIRT1, which promotes NRF2 dissociation from KEAP1 and facilitates its nuclear translocation. This process upregulates the expression of antioxidant enzymes, such as HO-1 (Singh and Ubaid, 2020). HO-1, a stress-induced isozyme, catalyzes heme degradation into biliverdin (BV), carbon monoxide (CO), and free iron (Fe^2+^) (Zhou et al., 2021). This process upregulates the expression of antioxidant enzymes, such as HO-1. For example, bilirubin, a product of biliverdin reduction, is a powerful antioxidant that scavenges ROS, thereby preventing protein and lipid peroxidation (Mcclements, 2020) (Graphical Abstract).

In summary, QGHJ contains various bioactive compounds, such as flavonoids, isoflavones, steroids, and monoterpenes, which synergistically affect bone metabolism, glucose homeostasis, and antioxidant defense via multiple signaling pathways and molecular targets. These coordinated actions may contribute to QGHJ’s overall therapeutic efficacy in managing T2DOP. However, some compounds may exhibit potential antagonistic effects. For example, although 17α-estradiol improves BMD, high concentrations of 17β-estradiol (10 µM) can severely disrupt cartilage formation in zebrafish, causing serious morphological defects (Fushimi et al., 2009). Moreover, high-dose 17β-estradiol treatment reduces osteoblast numbers in the craniofacial skeleton (Cohen et al., 2014), indicating that the bone formation-inhibiting effects of high-dose 17β-estradiol may counteract the bone formation-promoting effects of 17α-estradiol. The potential for other antagonistic interactions among QGHJ compounds requires further investigation to fully elucidate their net effects on T2DOP.

This study has several limitations that require further investigation. First, the use of rat and cell models limits the direct extrapolation of results to humans, necessitating validation through human clinical trials. Second, although QGHJ originates from traditional Chinese medicine and has a long history of use, its safety and potential side effects require thorough investigation. Future studies should evaluate the long-term safety of QGHJ. Furthermore, as the study was conducted in healthy adult rats, the evaluation of QGHJ’s effects in special populations is necessary. Moreover, any potential interactions with other medications commonly used by osteoporosis patients should be investigated. Finally, the synergistic and antagonistic effects among QGHJ compounds may significantly influence its pharmacological effects, emphasizing the need for further research on the interactions among these compounds and their impact on QGHJ’s overall efficacy.

## 5 Conclusion

In summary, the activation of the SIRT1/NRF2/HO-1 pathway and the nuclear translocation of NRF2 enhance antioxidative effects, which contribute to bone protection in T2DOP rats and elevate the vitality and osteogenic differentiation capacity of BMSCs.

## Data Availability

The original contributions presented in the study are included in the article/Supplementary Material. Further inquiries can be directed to the corresponding authors.

## Abbreviations

T2DOP, Type 2 diabetic ostecporosis; QGHJ, Qianggu concentrate; ELISA, Enzyme-Linked immunosorbent assay; BMSCs, Bone marrow mesenchymal stem cells; SIRT1, Silent information regulator 1; NRF2, Nuclear factor erythroid 2- related factor 2; HO-1, Heme oxygenase-1; RUNX2, Runt-Related transcription factor 2; PPARγ, Peroxisomeproliferator-activated receptor γ.
