# Supplementary material for: Qianggu concentrate: unlocking bone protection power via antioxidative SIRT1/NRF2/HO-1 pathways in type 2 diabetic osteoporosis
Source: Front Pharmacol. 2024 Aug 8;15:1426767. doi: 10.3389/fphar.2024.1426767 (PMC11338786; doi:10.3389/fphar.2024.1426767)
Supplement: Supplementary file 1 [file Table1.DOCX]

# Supplementary Table 1 Identification Results of Chemical Components in UPLC-HRMS.

| NO | RT (min） | Formula | m/z | Adduct | ppm | Score | Compound | SuperClass |
| --- | --- | --- | --- | --- | --- | --- | --- | --- |
| 1 | 2.97 | C11H11NO3 | 188.07 | [M+H-H2O]+ | 2.9 | 0.9704 | 3-Indolyllactic acid | Tryptophan alkaloids |
| 2 | 3.77 | C20H24NO4+ | 342.1688 | [M]+ | 3 | 0.9455 | Magnoflorine | Tyrosine alkaloids |
| 3 | 4.17 | C17H26O10 | 179.0696 | [M+H-C7H16O7]+ | 4.1 | 0.9932 | Loganin | Monoterpenoids |
| 4 | 4.26 | C25H30O13 | 197.0798 | [M+H-C15H18O9]+ | 3.3 | 0.9924 | Grandifloroside | Monoterpenoids |
| 5 | 4.6 | C11H8O4 | 205.0488 | [M+H]+ | 3.1 | 0.8497 | 3,7-Dihydroxy-2-naphthoic acid | Phenanthrenoids |
| 6 | 4.79 | C11H6O3 | 187.0383 | [M+H]+ | 2.3 | 0.9913 | Angecin | Coumarins |
| 7 | 5.85 | C25H24O13 | 533.1274 | [M+H]+ | 2.8 | 0.9936 | 6''-O-Malonylglycitin | Isoflavonoids |
| 8 | 6.96 | C12H16O4 | 207.1011 | [M+H-H2O]+ | 3.6 | 0.9922 | 6,7-Dihydroxyligustilide | Cyclic polyketides |
| 9 | 8.02 | C39H50O19 | 823.299 | [M+H]+ | 3.7 | 0.9337 | Epimedin C | Flavonoids |
| 10 | 8.24 | C33H40O15 | 677.2419 | [M+H]+ | 1.9 | 0.9831 | Icariin | Flavonoids |
| 11 | 8.52 | C11H6O3 | 187.0382 | [M+H]+ | 3.7 | 0.9954 | Ficusin | Coumarins |
| 12 | 8.88 | C11H6O3 | 187.0382 | [M+H]+ | 3.4 | 0.9955 | Bakuchicin | Coumarins |
| 13 | 9.55 | C30H48O4 | 437.34 | [M+H-2H2O]+ | 3.6 | 0.9597 | Hederagenol | Triterpenoids |
| 14 | 12.02 | C12H16O2 | 193.1215 | [M+H]+ | 3.6 | 0.9652 | Senkyunolide | Cyclic polyketides |
| 15 | 12.16 | C20H18O4 | 323.1265 | [M+H]+ | 3.7 | 0.956 | Neobavaisoflavone | Isoflavonoids |
| 16 | 12.29 | C20H20O4 | 325.1423 | [M+H]+ | 2.4 | 0.9413 | Bavachin | Flavonoids |
| 17 | 12.61 | C24H40O5 | 355.2619 | [M+H-3H2O]+ | 3.7 | 0.9829 | Cholan-24-oicacid,3,7,12-trihydroxy- | Steroids |
| 18 | 12.81 | C20H16O4 | 321.111 | [M+H]+ | 2.9 | 0.9969 | Corylin | Isoflavonoids |
| 19 | 12.93 | C12H14O2 | 191.106 | [M+H]+ | 3.8 | 0.9976 | Ligustilide A | Cyclic polyketides |
| 20 | 13.5 | C20H20O4 | 325.1423 | [M+H]+ | 2.9 | 0.9628 | Corylifolinin | Flavonoids |
| 21 | 13.63 | C21H22O4 | 339.1579 | [M+H]+ | 2.2 | 0.9847 | Bavachinin A | Flavonoids |
| 22 | 3.16 | C16H24O10 | 375.1283 | [M-H]- | 3.8 | 0.92 | Loganic acid | Monoterpenoids |
| 23 | 3.47 | C16H24O10 | 375.1285 | [M-H]- | 5.4 | 0.7737 | Mussaenosidic acid | Monoterpenoids |
| 24 | 3.65 | C16H18O9 | 353.0866 | [M-H]- | 3.9 | 0.9993 | Heriguard | Phenylpropanoids (C6-C3) |
| 3 | 4.16 | C17H26O10 | 435.1497 | [M+HCOO]- | 2 | 0.964 | Loganin | Monoterpenoids |
| 25 | 4.25 | C16H22O9 | 403.1235 | [M+HCO2]- | 2.8 | 0.9384 | Sweroside | Monoterpenoids |
| 26 | 4.59 | C17H18O9 | 365.0865 | [M-H]- | 3.5 | 0.9635 | (2E)-3-(4-(.beta.-D-Glucopyranosyloxy)-1-benzofuran-5-yl)prop-2-enoic acid | NA |
| 27 | 6.01 | C25H24O12 | 515.119 | [M-H]- | 0.6 | 0.9822 | 4,5-Di-O-caffeoylquinic acid | Phenylpropanoids (C6-C3) |
| 28 | 6.65 | C9H16O4 | 187.0974 | [M-H]- | 1.6 | 0.9984 | Azelaic acid | Fatty Acids and Conjugates |
| 29 | 7.99 | C31H35F3N8O3 | 659.2342 | [M+Cl]- | 20.8 | 0.8868 | CAY10626 | NA |
| 10 | 8.22 | C33H40O15 | 721.2344 | [M+HCO2]- | 0.1 | 0.9879 | Icariin | Flavonoids |
| 30 | 9.55 | C47H76O18 | 973.4988 | [M+HCOO]- | 2.6 | 0.9891 | Asperosaponin vi | Triterpenoids |
| 31 | 11.67 | C26H45NO7S | 514.2838 | [M-H]- | 1.2 | 0.9986 | Taurocholic acid | Steroids |
| 15 | 12.15 | C20H18O4 | 321.1127 | [M-H]- | 0.5 | 0.9991 | Neobavaisoflavone | Isoflavonoids |
| 32 | 12.27 | C20H20O4 | 323.1284 | [M-H]- | 0.9 | 0.9415 | Isobavachin | Flavonoids |
| 17 | 12.6 | C24H40O5 | 407.2791 | [M-H]- | 1.9 | 0.9998 | Cholic acid | Steroids |
| 18 | 12.8 | C20H16O4 | 319.0972 | [M-H]- | 0.5 | 0.9955 | Corylin | Isoflavonoids |
| 33 | 12.94 | C20H20O5 | 339.1225 | [M-H]- | 0.7 | 0.9587 | Licocoumarone | Isoflavonoids |
| 34 | 13.19 | C20H16O5 | 335.0921 | [M-H]- | 1.5 | 0.9857 | Psoralidin | Isoflavonoids |
| 35 | 13.77 | C26H45NO6S | 498.2888 | [M-H]- | 0.5 | 0.9986 | Taurohyodeoxycholic acid | Steroids |
| 36 | 14.58 | C18H24O2 | 271.1701 | [M-H]- | 0.4 | 0.8276 | .beta.-Estradiol | Steroids |
